# Genomic and biological control of *Sclerotinia sclerotiorum* using an extracellular extract from *Bacillus velezensis* 20507

**DOI:** 10.3389/fmicb.2024.1385067

**Published:** 2024-03-26

**Authors:** Yunqing Cheng, Hanxiao Lou, Hongli He, Xinyi He, Zicheng Wang, Xin Gao, Jianfeng Liu

**Affiliations:** ^1^Jilin Provincial Key Laboratory of Plant Resource Science and Green Production, Jilin Normal University, Siping, Jilin, China; ^2^Department of Microbiology, Oregon State University, Corvallis, OR, United States

**Keywords:** *Bacillus velezensis*, *Sclerotinia sclerotiorum*, biological control, whole genome sequencing, antagonism

## Abstract

**Introduction:**

*Sclerotinia sclerotiorum* is a known pathogen that harms crops and vegetables. Unfortunately, there is a lack of effective biological control measures for this pathogen. *Bacillus velezensis* 20507 has a strong antagonistic effect on *S. Sclerotiorum*; however, the biological basis of its antifungal effect is not fully understood.

**Methods:**

In this study, the broad-spectrum antagonistic microorganisms of *B. velezensis* 20507 were investigated, and the active antifungal ingredients in this strain were isolated, purified, identified and thermal stability experiments were carried out to explore its antifungal mechanism.

**Results:**

The *B. velezensis* 20507 genome comprised one circular chromosome with a length of 4,043,341 bp, including 3,879 genes, 185 tandem repeats, 87 tRNAs, and 27 rRNAs. Comparative genomic analysis revealed that our sequenced strain had the closest genetic relationship with *Bacillus velezensis* (GenBank ID: NC 009725.2); however, there were significant differences in the positions of genes within the two genomes. It is predicted that *B. velezensis* 20507 encode 12 secondary metabolites, including difficidin, macrolactin H, fengycin, surfactin, bacillibactin, bacillothiazole A-N, butirosin a/b, and bacillaene. Results showed that *B. velezensis* 20507 produced various antagonistic effects on six plant pathogen strains: *Exserohilum turcicum, Pyricularia oryzae, Fusarium graminearum, Sclerotinia sclerotiorum, Fusarium oxysporum*, and *Fusarium verticillioides*. Acid precipitation followed by 80% methanol leaching is an effective method for isolating the antifungal component ME80 in *B. velezensis* 20507, which can damage the membranes of *S. sclerotiorum* hyphae and has good heat resistance. Using high-performance liquid chromatography, and Mass Spectrometry analysis, it is believed that fengycin C72H110N12O20 is the main active antifungal substance.

**Discussion:**

This study provides new resources for the biological control of *S. Sclerotiorum* in soybeans and a theoretical basis for further clarification of the mechanism of action of *B. velezensis* 20507.

## 1 Introduction

Bacillus spp. are plant growth promoting rhizobacteria (PGPR) that promote plant growth, absorb and utilize mineral nutrients, and inhibit harmful organisms. Bacillus spp. are also the most studied and applied group of biocontrol bacteria and are characterized by broad-spectrum efficiency, easy cultivation, stress tolerance, and storage tolerance. Secondary metabolites are important for the biocontrol of Bacillus spp. (Djordje et al., 2018; Santos et al., 2023; Zhang et al., 2023). It has been found that Bacillus spp. produce various secondary metabolites beneficial to plants, including lipopeptide compounds (Chen et al., 2009) synthesized by non-ribosome peptide synthesis (NRPS) and polyketide compounds synthesized by polyketide synthase (PKS) (Ruckert et al., 2011), and linear azol (In) E-containing peptides (LAP) (Baniulis, 2021), bacteriocin (Diep and Nes, 2002), thiopeptide (Bleich et al., 2015), and terpene (Kontnik et al., 2008) synthesized by ribosome peptide synthesis (RPS). Various beneficial secondary metabolites are secreted by Bacillus spp., and whole-genome sequencing is important for understanding and utilizing biocontrol strains.

*Sclerotinia sclerotiorum* is a common disease in soybean crops that mainly causes stem rot and spreads quickly (Cheng et al., 2022; Liu J. et al., 2022). In severe cases, it can lead to crop failure (Chen et al., 2005). *B. amyloliquefaciens* CH-2 inhibits the growth of *S. sclerotiorum*, but also inhibited the formation of sclerotia. When managing the disease during the crop production cycle, the main form of control is through synthetic fungicides (Rocha et al., 2023). The biocontrol strain *Bacillus velezensis* was isolated from our laboratory. *B. velezensis* transports antifungal substances out of the cell and has a strong antagonistic effect on the pathogenic microorganism *S. sclerotiorum* in pot experiments. It can inhibit the expression of genes encoding the ribosomal subunit of *S. sclerotiorum*, indicating its potential for biocontrol applications. Owing to the high degree of genetic conservation, physiological analysis of the 16S rRNA gene cannot differentiate between *B. velezensis* and *B. amyloliquefaciens* (Chun et al., 2019; Wang et al., 2022). Therefore, we previously believed that the biocontrol bacterium, *B. velezensis*, was *B. amyloliquefaciens*. Currently, the antifungal substances produced by this strain are mainly secreted outside the cell. We found that this strain inhibited the expression of genes encoding the ribosomal subunit of *S. scrotum*, resulting in the inhibition of protein synthesis. As a biocontrol bacterium with potential application value, many characteristics need to be explored further, including genomic sequences and their annotations, potential metabolite, the broad-spectrum ability to inhibit fungi, detailed active ingredients for inhibiting fungi and their thermal stability, etc. A deeper understanding of *B. velezensis* is crucial for its application in production.

In this study, we sequenced the entire genome of *B. velezensis* and predicted the types of secondary metabolites it produces based on genetic information (NCBI accession no. PRJNA981422). Furthermore, purification of extracellular products was carried out using various methods, such as ammonium sulfate precipitation, and research was conducted on the broad-spectrum properties and thermal stability of the antifungal components. This study provides a theoretical basis for the utilization of *B. velezensis* in practice.

## 2 Results

### 2.1 Genome sequencing, assembly, and gene annotation

**FIGURE 1:**
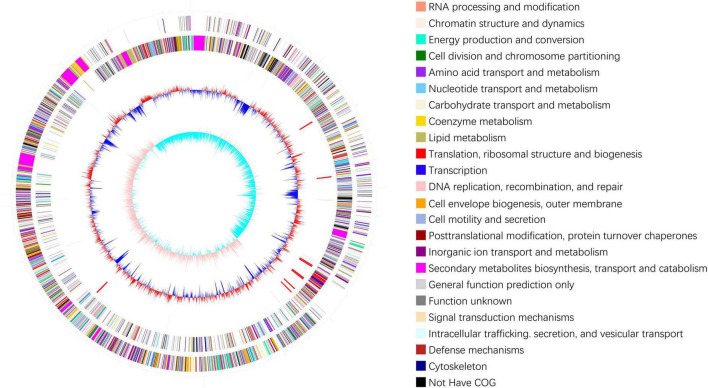
Genomic cycle diagram of *Bacillus velezensis* 20507. The outermost circle of the circle chart is an indicator of genome size, with each scale being 0.5 Mb. The second and third circles represent CDS on positive and negative chains, with different colors indicating the functional classification of different COGs in CDS. The fourth circle contains rRNA and tRNA. The fifth circle shows the GC content. The innermost circle is the GC skew value.

In total, 12,592,676 raw reads with lengths of 1,888,901,400 bp were obtained by Illumina sequencing. After filtering low-quality reads, 12574834 clean reads with lengths of 1,828,325,506 bp and 46.17% GC content were acquired. After sequence assembly, the genome of *B. velezensis* 20507 was found to comprise one circular chromosome with a length of 4,043,341 bp and 46.34% GC content, including 3,879 genes, 185 tandem repeats, 87 tRNAs, and 27 rRNAs (Figure 1). The main parameters of the genes in the genome were as follows: the total length of the genes was 3582372 bp, with an average length of 923 bp for each gene. Gene and intergenic lengths accounted for 88.60 and 11.40% of the genome, respectively.

**FIGURE 2:**
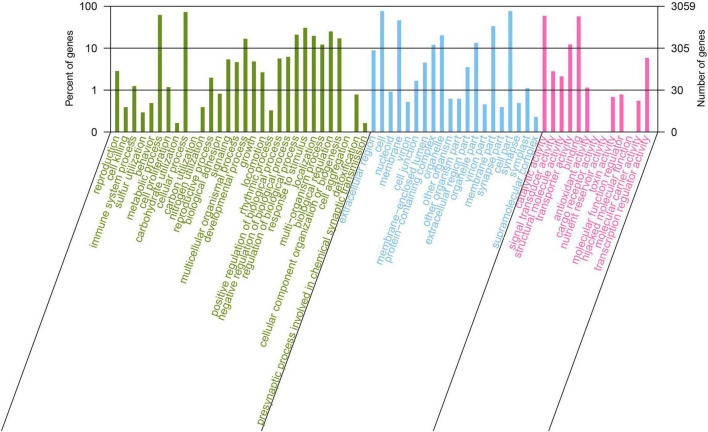
Gene Ontology annotation of *Bacillus velezensis* 20507 genome.

**FIGURE 3:**
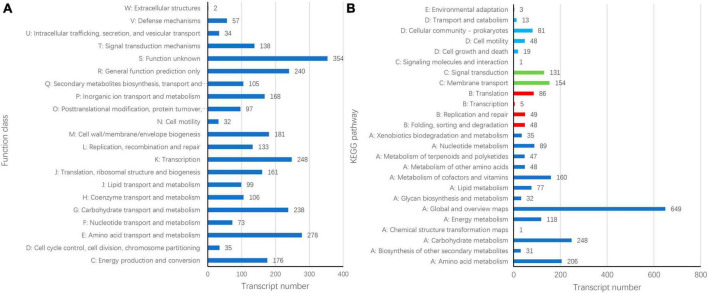
Cluster of orthologous groups of proteins [COG, **(A)**] and Kyoto encyclopedia of genes and genomes **(B)** annotation of *Bacillus velezensis* 20507 genome.

Gene Ontology (GO) annotation analysis was conducted on the BLAST results using the blast2go software, and 3,059 genes were annotated. GO annotations included three subcategories: biological processes (30 branches), cellular components (20 branches), and molecular functions (13 branches). In the subcategories of biological processes, the three largest branches were cellular processes (GO:0009987), metabolic processes (GO:0008152), and responses to stimuli (GO:0050896) (Figure 2). Among the cellular components subcategories, the three largest branches were cells (GO:0005623), cell parts (GO:0044464), and membranes (GO:0016020) (Figure 2). In the molecular function subcategory, the three largest branches were catalytic (GO:0003824), binding (GO:0005488), and transporter activities (GO:0005215) (Figure 2). Among the 3059 identified genes, 2250 and 2904 were annotated using the KEGG and COG functional databases, respectively. For the COG categories, the number of genes with unknown (354) function was the highest, followed by amino acid transport and metabolism (278), transcription (248), general function prediction only (240), and carbohydrate transport and metabolism (238); the number of other subcategories was less than 200 (Figure 3A). The KEGG pathway with the highest number of genes was global and overview maps (649), followed by carbohydrate metabolism (248), amino acid metabolism (206), metabolism of cofactors and vitamins (160), membrane transport (154), signal transduction (131), and energy metabolism (118); the number of other subcategories was less than 100 (Figure 3B).

### 2.2 Comparative genomics analysis

**FIGURE 4:**
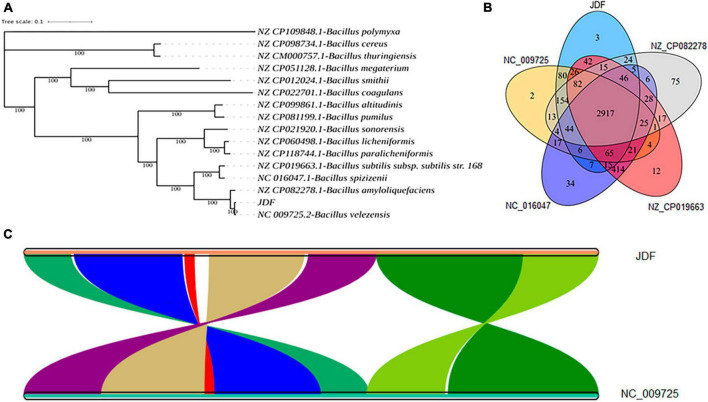
Comparative genomics analysis of *Bacillus velezensis* 20507. **(A)** Phylogenetic analysis of *Bacillus velezensis* 20507. **(B)** Venn diagram based on gene family analysis of *Bacillus velezensis* 20507 and four nearby species. **(C)** Collinearity analysis of at gene level. **(C)** The lines in the figure represent the positional connections of homologous genes between two species, and the colors do not represent specific meanings. Colored areas have a span greater than 100 in the contiguous area. JDF, *Bacillus velezensis* 20507; NC_009725.2, *Bacillus velezensis*; NZ_CP082278.1, *Bacillus amyloliquefaciens*; NZ_CP019663.1, *Bacillus subtilis* subsp. subtilis str. 168; NC_016047.1, *Bacillus spizizenii*.

The phylogenetic tree of 15 closely related species revealed that the sequenced strain JDF had the closest genetic relationship with *Bacillus velezensis* (Gene bank ID: NC 009725.2), followed by *Bacillus amyloliquefaciens* (Gene bank ID: NZ CP082278.1), *Bacillus subtilis* subsp. subtilis str. 168 (NZ CP019663.1), and *Bacillus spizizenii* (NC 016047.1), and had a distant genetic relationship with *Bacillus polymyxa* (NZ CP109848.1), *Bacillus cereus* (NZ CP098734.1), and *Bacillus thuringiensis* (NZ CM000757.1) (Figure 4A). Therefore, we believe that our sequenced strain, JDF, belongs to *Bacillus velezensis*.

JDF and its four nearby species had 3879, 3689, 4085, 4278, and 3872 genes, respectively (Table 1). When all genes were clustered into orthogroups using Orthofinder software, these five species obtained 3531, 3461, 3654, 3730, and 3456 orthogroups (Table 1), respectively. These five nearby species shared 2917 orthogroups (Figure 4B), accounting for 78–84% of their respective orthogroups. The number of specific orthogroups in our sequenced strain JDF was three, which was far less than the 75 and 34 of *Bacillus amyloliquefaciens* (NZ_CP082278) and *Bacillus spizizenii* (NC_016047) (Table 1), respectively. JDF and *Bacillus velezensis* (NC_009725) shared 3374 orthogroups (Figure 4B), accounting for 96 and 97% of their respective orthogroups, respectively. This confirmed a close genetic relationship between the two strains, which was consistent with the results of genetic evolution analysis (Figure 4A).

**TABLE 1:** Classification and statistics of gene families.

Statistical indicators	Species name				
	**JDF**	**NC_009725**	**NC_016047**	**NZ_CP019663**	**NZ_CP082278**
Number of genes	3879	3689	4085	4278	3872
Number of genes in orthogroups	3727	3630	3980	4001	3727
Number of unassigned genes	152	59	105	277	145
Percentage of genes in orthogroups	96.1	98.4	97.4	93.5	96.3
Percentage of unassigned genes	3.9	1.6	2.6	6.5	3.7
Number of orthogroups containing species	3531	3461	3654	3730	3456
Percentage of orthogroups containing species	84	82.3	86.9	88.7	82.2
Number of species-specific orthogroups	3	2	34	12	75
Number of genes in species-specific orthogroups	13	4	71	25	164
Percentage of genes in species-specific orthogroups	0.3	0.1	1.7	0.6	4.2

NC_009725.2, *Bacillus velezensis*; NZ_CP082278.1, *Bacillus amyloliquefaciens*; NZ_CP019663.1, *Bacillus subtilis* subsp. subtilis str. 168; NZ_016047.1, *Bacillus spizizenii*. Number of genes: the total number of genes in the strain; Number of genes in orthogroups: the number of genes in a gene family; Number of unassigned genes: the number of genes that cannot be clustered with other genes; Percentage of genes in orthogroups: the proportion of sample genes in the gene family; Percentage of unassigned genes: the proportion of genes in a species that cannot be clustered with other genes. Number of orthogroups containing specifications: the number of gene families in the sample; Percentage of orthogroups containing species: the proportion of gene families in the sample; Number of species-specific orthogroups: number of specific gene families in the strain; Number of genes in species-specific orthogroups: gene number of specific gene families in the strain; Percentage of genes in species-specific orthogroups: the proportion of genes in the unique gene family of the strain.

Average Nucleotide Identity (ANI) is an indicator of the phylogenetic relationship between two genomes at the nucleotide level. ANI is defined as the average base similarity between homologous fragments of two microbial genomes and is characterized by a high degree of discrimination between closely related species. The ANI of the JDF and *Bacillus velezensis* (NC_009725) genomes was 98%, suggesting a high sequence similarity. Collinearity analysis of these two genomes was conducted using MCScan software, and the results confirmed that the sequences of the two genomes were highly similar, but there were significant differences in the positions of genes within the genome (Figure 4C).

### 2.3 Metabolite prediction

Through online prediction using anti-SMASH and alignment analysis with NCBI BLAST, it was found that *B. velezensis* 20507 encodes 12 secondary metabolite synthesis gene clusters, of which nine gene clusters had similar known clusters and three gene clusters had no similar known clusters (Table 2). The length of the 12 secondary metabolite synthesis gene clusters was 735,348 bp (Table 2), accounting for 18.19% of the *B. velezensis* 20507 genome. It was predicted that *B. velezensis* 20507 encodes nine secondary metabolites, including difficidin and macrolactin H synthesized via the polyketide pathway; fengycin, surfactin, bacillibactin, and bacillothiazol A-N synthesized through the NRP pathway; butirosin a/butirosin b synthesized through the saccharide pathway; and bacillaene synthesized through the Polyketide + NRP pathway. Compared to known gene clusters, the similarity between difficidin, fengycin, bacillaene, macrolactin H, bacilysin, bacillibactin, and bacillothiazole A-N gene clusters was 100%. The alignment between the gene clusters encoding butirosin and surfactin was low at 7 and 82%, respectively. In addition, *B. velezensis* 20507 may encode three unknown secondary metabolites, initially predicted to be one polyketide and two terpene compounds.

**TABLE 2:** Predicted secondary metabolite synthesis gene cluster in genome of *Bacillus velezensis* 20507.

From–To	Type	Most similar known cluster			
		**Products**	**Pathways**	**Similarity/%**	**Resources**
63,384 to 155,781	TransAT-PKS	Difficidin	Polyketide	100	*Bacillus velezensis* FZB42
285,047 to 325,740	T3PKS	–	–	–	–
401,872 to b421,998	Terpene	–	–	–	–
449,338 to 582,324	NRPS, betalactone, transAT-PKS, RIPP-like	Fengycin	NRP	100	*Bacillus velezensis* FZB42
666,832 to 767,429	transAT-PKS, NRPS, T3PKS	Bacillaene	Polyketide + NRP	100	*Bacillus velezensis* FZB42
990,930 to 1,077,307	TransAT-PKS	Macrolactin H	Polyketide	100	*Bacillus velezensis* FZB42
1,458,763 to 1,476,170	Terpene	–	–	–	–
1,562,435 to 1,601,681	PKS-like	Butirosin A/butirosin B	Saccharide	7	*Bacillus circulans*
2,160,103 to 2,225,184	NRPS	Surfactin	NRP: lipopeptide	82	*Bacillus velezensis* FZB42
2,834,105 to 2,875,523	Other	Bacilysin	Other	100	*Bacillus velezensis* FZB42
3,397,975 to 3,447,479	RiPP-like, NRP-metallophore, NRPS	Bacillibactin	NRP	100	*Bacillus subtilis* subsp. subtilis str. 168
3,552,453 to 3,601,969	NRPS	Bacillothiazol A-N	NRP	100	*Bacillus velezensis* FZB42

PKS, polyketide synthase; NRPS, non-ribosomal peptide synthase; NRP, non-ribosomal peptide.

### 2.4 Antagonistic effects of *B. velezensis* 20507 on six plant pathogenic fungi

**FIGURE 5:**
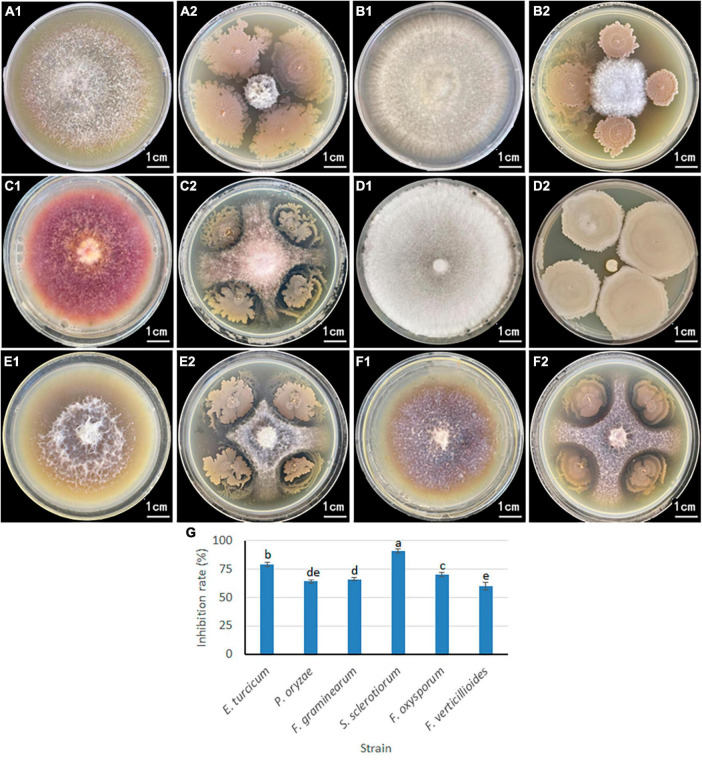
Inhibitory effect of antagonistic bacteria *Bacillus velezensis* 20507 on growth of six plant pathogens. For **(A2,B2,C2,D2,E2,F2)**, the plant pathogenic fungi were inoculated in the center of the Petri dish, and the biocontrol bacteria were inoculated on the four corners of the pathogenic fungi. **(A1)** *Exserohilum turcicum*; **(A2)** *Exserohilum turcicum* and *Bacillus velezensis 20507*; **(B1)** *Pyricularia oryzae*; **(B2)** *Pyricularia oryzae* and *B. velezensis 20507*; **(C1)** *Fusarium graminearum*; **(C2)** *Fusarium graminearum* and *Bacillus velezensis 20507*; **(D1)** *Sclerotinia sclerotiorum*; **(D2)** *Sclerotinia sclerotiorum* and *B. velezensis 20507*; **(E1)** *Fusarium oxysporum*; **(E2)** *Fusarium oxysporum* and *B. velezensis 20507*; **(F1)** *Fusarium verticillioides*; **(F2)** *Fusarium verticillioides* and *B. velezensis 20507*. **(G)** Inhibition rate of *B. velezensis* 20507 against six pathogenic fungi. In the bar chart, different lowercase letters above the columns indicate significant differences at *P* = 0.05 level.

A plate confrontation experiment was conducted to determine the relationship between *B. velezensis* 20507 and six pathogenic plant fungi (Figure 5): *Exserohilum turcicum*, *Pyricularia oryzae*, *Fusarium graminearum*, *Sclerotinia sclerotiorum*, *Fusarium oxysporum*, and *Fusarium verticillioides.* Among these, S. sclerotiorum and E. turcicum had the smallest colonies, and these two pathogenic fungus grew only in a small area in the center of the plate (Figures 5A1, A2, D1, D2). It is believed that *B. velezensis* 20507 strongly inhibit the growth of *S. sclerotiorum* and *E. turcicum*. The colony sizes of *P. oryzae* (Figures 5B1, B2), *F. graminearum* (Figures 5C1, C2), *F. oxysporum* (Figures 5E1, E2), and *F. verticillioides* (Figures 5F1, F2) were relatively large, suggesting that *B. velezensis* 20507 could also effectively inhibit the growth of these four fungi. The colony radii of all six pathogenic fungi in the antagonistic experiment were measured and the inhibition rate was calculated. The results showed that the inhibition rate of *B. velezensis* 20507 against six pathogens was more than 60%. *S. sclerotiorum* and *E. turcicum* showed the highest inhibition rates of 91 and 79%, respectively, which were significantly higher than those of the other four pathogenic fungi (*P* < 0.05) (Figure 5G). These results showed that when inoculated with *B. velezensis* 20507, the biocontrol bacteria showed varying degrees of inhibitory effects on the growth of all six fungi.

### 2.5 Inhibitory effect of crude solution on the growth of *S. sclerotiorum*

**FIGURE 6:**
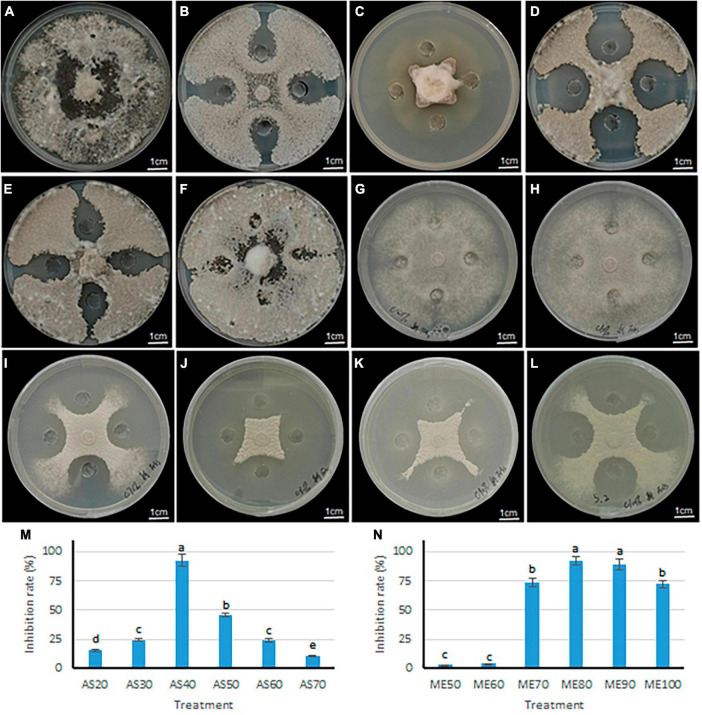
Inhibitory effect of antagonistic bacteria *B. velezensis* 20507 on growth of six plant pathogens. **(A–F)** The inhibitory effects of crude extract from different ammonium sulfate saturation conditions on the growth of *S. sclerotiorum*. **(A–F)** represents ammonium sulfate saturation of 20, 30, 40, 50, 60, and 70%, respectively. **(G–L)** The inhibitory effects of crude extract from different concentration methanol leaching conditions on the growth of *S. sclerotiorum*. **(G–L)** Represents methanol concentration of 50, 60, 70, 80, 90, and 100%. **(M)** Inhibition rate of crude extract from *B. velezensis* 20507 against *Sclerotinia sclerotiorum* using different ammonium sulfate saturation conditions. AS20, AS30, AS40, AS50, AS60, and AS70 represents ammonium sulfate saturation of 20, 30, 40, 50, 60, and 70%, respectively. **(N)** Inhibition rate of crude extract from *B. velezensis* 20507 against *Sclerotinia sclerotiorum* using different concentration methanol leaching conditions. ME50, ME60, ME70, ME80, ME90, and ME100 represents 50, 60, 70, 80, 90, and 100% methanol leaching conditions, respectively. In the bar chart, different lowercase letters above the columns indicate significant differences at *P* = 0.05 level.

Two methods of ammonium sulfate precision and methanol leaching were used to purify the antimicrobial components in the fermentation broth of *B. velezensis* 20507. The differences in antimicrobial activity of precipitates under different ammonium sulfate saturation conditions and different concentrations of methanol leaching were evaluated. Within the ammonium sulfate saturation range of 20–40%, as ammonium sulfate saturation increased, the colony diameter of *S. sclerotiorum* decreased (Figures 6A–C). However, when the saturation exceeded 40%, the diameter of inhibition zone decreases with the increase of ammonium sulfate saturation at 50–70% (Figures 6D–F). Consistent with these, the inhibition rate of AS40 against *S. sclerotiorum* was significantly higher than the other five treatments (*P* < 0.05) (Figure 6M). These results suggest that 40% ammonium sulfate saturation is most favorable for the precipitation of antimicrobial components.

The antifungal activity of sediments under different concentrations of methanol was evaluated, and the results showed that the diameter of the *S. sclerotiorum* colony decreased with the increase of methanol concentration in the range of 50–80% (Figures 6G–J). However, in the range of 90–100%, the diameter of the *S. sclerotiorum* colony increased with the increase of methanol concentration (Figures 6K, L). Consistent with these, the inhibition rate of ME80 and ME90 against *S. sclerotiorum* was significantly higher than the other four treatments (*P* < 0.05) (Figure 6N). Among all six treatments, the ME80 treatment had the highest inhibition rate. Therefore, 80% methanol concentration is most conducive to the precipitation of antifungal substances. In summary, we further purified and identified the antifungal substances using sediments obtained by 40% ammonium sulfate saturation (AS40) and 80% methanol leaching (ME80).

### 2.6 Effect of ME80 treatment on the integrity of *S. sclerotiorum* cell membranes

**FIGURE 7:**
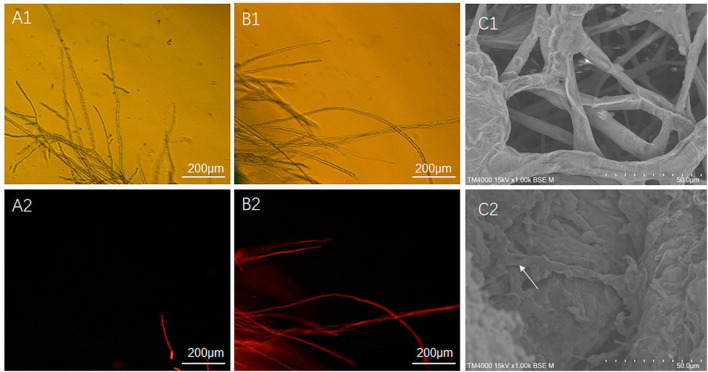
Effect of 80% methanol leaching treatment on the integrity of *S. sclerotiorum* cell membrane. **(A1)** Control hypha of *S. sclerotiorum*, observed with fluorescence microscope under visible light conditions. **(A2)** Control hypha of *S. sclerotiorum*, observed with fluorescence microscope under fluorescence conditions. **(B1)** Treatment hypha of *S. sclerotiorum*, observed with fluorescence microscope under visible light conditions. **(B2)** Treatment hypha of *S. sclerotiorum*, observation with fluorescence microscope under fluorescence conditions. **(C1)** Control hypha of *S. sclerotiorum*, observed with scanning electron microscope. **(C2)** Treatment hypha of *S. sclerotiorum*, observed with scanning electron microscope. White Arrows indicate damaged hyphae. Control, hypha of *S. sclerotiorum* was treated with distilled water for 20 min. Treatment, hypha of *S. sclerotiorum* was treated with 100 μg/mL ME80 aqueous solution for 20 min. An excitation wavelength was 535 nm.

In this study, two methods were used for the separation and purification of antimicrobial substances, including ammonium sulfate prediction and acid prediction followed by methanol leaching. The amount of antimicrobial substances obtained using the latter method was approximately ten times higher than that obtained using the former method. The difference in antimicrobial effects between the two initial extracts was tested, and it was found that the antimicrobial effects were similar. Therefore, we focused on further analysis and testing of the antimicrobial substance (ME80) obtained using the latter method. ME80 aqueous solution was used to treat the mycelium of *S. sclerotiorum*, and PI staining was performed to observe damage to the cell membrane. PI can penetrate the cell membranes of dead cells and stain the nucleus red. However, PI cannot penetrate the membranes of living cells. After being dyed with PI, most control mycelia in *S. sclerotiorum* did not stain, and only a few mycelia were stained red by PI (Figures 7A1, A2). The red color was believed to be caused by physical damage to a single mycelium during the staining and washing processes, indicating that the control mycelial structure was generally intact. ME80 aqueous solution was used to treat *S. sclerotiorum* mycelia for 20 min, and all mycelia were stained red with PI (Figures 7B1, B2), indicating that ME80 treatment led to damage to the *S. sclerotiorum* mycelium. The damage to the control mycelia and ME80 aqueous solution mycelia was further observed using scanning electron microscopy, and the results were consistent with those observed under fluorescence microscopy (Figures 7C1, C2). In summary, ME80 treatment can damage *S. sclerotiorum*. Therefore, the ME80 extract mixture should contain antimicrobial components that cause cell membrane leakage in *S. sclerotiorum*.

### 2.7 Isolation and identification of antifungal components

**FIGURE 8:**
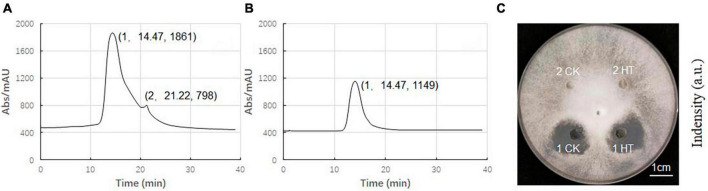
High performance liquid chromatography (HPLC) analysis of antibacterial components in 80% methanol leaching of *Bacillus velezensis* 20507. **(A)** HPLC spectrum of ME80 of *Bacillus velezensis* 20507. **(B)** The spectrum of component 1 collected, and component 2 has been removed. **(C)** Plate antagonism experiment using obtained component 1 and component 2; 1 CK, component 1 at ordinary temperature; 1HT, component 1 was heated at 121°C for 20 min; 2CK, component 2 at ordinary temperature; 2HT, component 2 was heated at 121°C for 20 min. HPLC, high-performance liquid chromatography; ME80, Acid precipitation followed by 80% methanol leaching.

**FIGURE 9:**
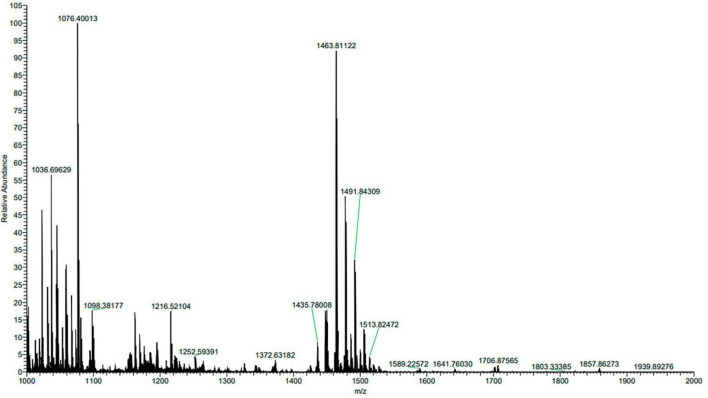
Triple TOF-MS/MS analysis of main antibacterial in *Bacillus velezensis* 20507. Component 1 in ME80 was used as a sample. Using liquid chromatography columns, the substance collection solution from 12 to 15 min was mixed to obtain component 1.

The composition of ME80 was analyzed using HPLC. After separation using a chromatographic column, two components were obtained with retention times of 14.47 and 21.22 min, respectively. Among them, the peak area corresponding to the first chemical component was 2.33 times that of the second peak area (Figure 8A). We mixed the substance collection solution from 12 to 15 min to obtain component 1 and conducted the HPLC analysis again. These results confirm that pure component 1 was obtained (Figure 8B). We mixed the substance collection solutions for 21–23 min to obtain component 2. Antagonistic experiments were conducted using components 1 and 2 against *S. sclerotiorum*, confirming that component 1 indeed has antifungal activity (Figure 8C). Moreover, the chemical stability of component 1 is particularly good, and after high-pressure sterilization at 121°C, the antifungal activity does not decrease at all (Figure 8C). Triple TOF-MS analysis was performed on component 1, and the mass-to-charge ratio of component 1 was 1463.8, corresponding to a molecular weight of 1463.8 Da, which was derived from the molecular weight of fengycin C_72_H_110_N_12_O_20_ (Yu et al., 2024; Figure 9).

## 3 Discussion

Through a series of analyses, including genome sequencing, prediction of secondary metabolites, precipitation of antifungal substances in the fermentation broth, analysis of antifungal activity, HPLC purification, and mass spectrometry identification of antifungal components, the results suggested that the main antifungal component in *Bacillus velezensis* 20507 was fengycin. Fengycin cyclic lipopeptides contain a series of homologs (Honma et al., 2012), and are broad-spectrum antifungal preparations that are particularly effective against filamentous fungi (Vanittankom et al., 1986; Koumoutsi et al., 2004; Ongena and Jacques, 2008; Villegas-Escobar et al., 2013). They have antagonistic effects on pathogenic fungi in rapeseed and wheat, and are recommended for use in agriculture (Ramarathnam et al., 2007). Fengycin is synthesized by five non-ribosomal fengycin synthase enzymes: FenC, FenD, FenE, FenA, and FenB (Devine and Hancock, 2002; Zasloff, 2002). Fengycins exert anti-fungal effects by disrupting the cell membrane and damaging the cell structure, and the selectivity of this function is related to the composition of fungal cell membranes. The comparative genomic results indicated that the strain we isolated was *Bacillus velezensis*, which has 98% sequence similarity with the reported strain *Bacillus velezensis* (NC:009725), but there were significant differences in the gene arrangement order. Through anti SMASH and alignment analysis with NCBI BLAST, it was found that strain *Bacillus velezensis* 20507 might encode a 12 secondary metabolite synthesis gene cluster, which includes all genes of the fengycin synthase gene family: FenC, FenD, FenE, FenA, and FenB (Supplementary Table 1). Some studies have suggested that the components related to fengycin synthase are arranged in a modular manner, in the order of FenC-FenD-FenE-FenA-FenB, and exhibit collinearity with the arrangement of amino acid residues in fengycins (Wu et al., 2007). In this study, tig00000001_ Pilon_ 468, tig00000001_ Pilon_ 469, tig00000001_ Pilon_ 470, tig00000001_ Pilon_ 471, and tig00000001_pilon_472 sequentially encode five genes, including FenA, FenB, FenC, FenD, and FenE. This implied that there was a significant difference in the amino acid residue arrangement between the fengycins of our strain and the reported *Bacillus velezensis* FZB42 fengycins (Hanif et al., 2019). When fengycins bind to the fungal cell membrane, they form large aggregates, disrupting the normal ordering of phospholipid molecules on the cell membrane and causing cytoplasmic efflux, resulting in cell death (Sur et al., 2018). Similar to other lipopeptide antibiotics, fengycins exhibit broad-spectrum antifungal activity, low toxicity, and low susceptibility to drug resistance. They are a new type of antibiotics with developmental potential in medical, agricultural, and animal husbandry production (Medeot et al., 2020). Our results indicate that *Bacillus velezensis* 20507 can also cause cell membrane damage, which is consistent with a previous study (Sur et al., 2018). Generally, antibiotics are resistant to high temperatures. If antibiotics are added to the culture medium for plant or microbial cultivation, when the temperature of the culture medium is low, they must be filtered and sterilized before being added. Astonishingly, fengycins produced by *Bacillus velezensis* 20507 retained antifungal activity after high-temperature sterilization at 121°C. This characteristic is far superior to that of ordinary antibiotics, and it is believed that this ingredient has broad application prospects in the prevention and control of plant pathogens, food additives, and even in the medical field.

## 4 Materials and methods

### 4.1 Experimental strain

The experimental *B. velezensis* strain was isolated and purified in our laboratory and is now stored at the China General Microbiological Culture Collection Center under strain number 20507. Six plant pathogenic fungi were also used to test the antifungal substances of *B. velezensis* 20507, including: *Exserohilum turcicum*, *Pyricularia oryzae*, *Fusarium graminearum*, *Sclerotinia sclerotiorum*, *Fusarium oxysporum*, and *Fusarium verticillioides*. The plant pathogens were isolated and preserved in our laboratory.

### 4.2 Genome sequencing

*Bacillus velezensis* 20507 was cultured in LB medium at 37°C, at 200 r/min, for 12 h. After centrifugation at 5000 × *g* for 10 min at 4°C, bacteria were collected, and the total genomic DNA was extracted. Genomic DNA was collected using 1% agarose gel electrophoresis. High purity DNA samples were sent to Shanghai Yuanshen Biomedical Technology Co., Ltd. for sequencing analysis. First, a Covaris M220 Focused Ultrasonicator (COVARIS, INC) was used for genomic DNA fragmentation (300–500 bp). Then, TruSeq™ DNA Sample Prep Kit DNA (Illumina Inc., San Diego, CA, USA) was used to construct a sequencing library. Bridge PCR amplification of the sequencing library was performed using TruSeq PE Cluster Kit v3-cBot-HS (Illumina). Finally, the bridge PCR amplification products were preprocessed using the Truseq SBS Kit v3-HS (200cycles) (Illumina), followed by sequencing on the Illumina NovaSeq 6000 platform.

### 4.3 Genome assembly and gene annotation

Correct and trim software in Canu (v1.6)^1^ (Koren et al., 2017) was used to correct bases and trim low-quality regions in the sequencing reads. The assembly software in Canu (v1.6) was used to perform genome sequence splicing. Racon (v1.4.20)^2^ and Pilon (v1.22)^3^ were used to polish the sequences and generate the final corrected sequence. Quast (v5.1.0rc1)^4^ was used to evaluate the assembly results. The genome sequence of *B. velezensis* 20507 was submitted to the NCBI (National Center for Biotechnology Information) GenBank under the accession number PRJNA981422. Circos software (v0.64)^5^ (Krzywinski et al., 2009) was used to draw the genome loop diagram, and the protein sequences of the predicted genes were aligned to the Nr, Genes, String, and Gene Ontology (GO) databases to obtain annotation information using blastp (BLAST 2.2.28 +). Barrnap (v0.4.2)^6^ and tRNAscan-SE (v1.3.1)^7^ (Lowe and Eddy, 1997) were used to predict rRNA and tRNA in the genome of *B. velezensis* 20507 respectively. RepeatMasker (v4.0.7) (Tarailo-Graovac and Chen, 2009)^8^ was used to predict the repeat sequences. Prodigal (v2.6.3) (She et al., 2009)^9^ was used to predict genes in the genome.

### 4.4 Comparative genomics analysis

(1) Phylogenetic analysis: After downloading protein sequences from 15 species, homologous gene analysis was performed using Orthofinder software (Emms and Kelly, 2019). To avoid interference from collateral homologous proteins, homologous genes for which all 15 species had a single copy were selected to perform gene multiple sequence alignment and construct a single copy gene matrix using MUSCLE v3.7 software,^10^ followed by the construction of a species phylogenetic tree using RAxml software. (2) Gene family analysis: Based on the above phylogenetic tree, four nearby species were identified and selected for further gene family analysis, including *Bacillus velezensis*, *Bacillus amyloliquefaciens*, *Bacillus subtilis* subsp. subtilis str. 168, and *Bacillus spizizenii*. OrthoFinder software was used to classify the predicted protein sequences of the sequenced strains and protein sequences of the reference genome into families. Then, the gene families were subjected to further analysis, and information including the unique gene families of the strains, common gene families of the strains, and single-copy gene families of each strain was obtained. Finally, a Venn diagram was constructed using the statistical results for the gene families. (3) Collinearity analysis: Genome average nucleotide consistency analysis between our sequenced genome and *Bacillus velezensis* (NC_009725.2) was performed using FastANI software. MCScan software was used to draw collinearity diagrams based on collinearity relationships.

### 4.5 Prediction of secondary metabolic gene cluster

Anti-SMASH (v6.0.1)^11^ software was used to predict secondary metabolites in the genome of *B. velezensis* 20507 (Blin et al., 2021).

### 4.6 Plate confrontation experiment

To determine whether *B. velezensis* 20507 has broad antifungal effects, plate confrontation experiments were conducted. Six plant pathogens (*Exserohilum turcicum*, *Pyricularia oryzae*, *Fusarium graminearum*, *Sclerotinia sclerotiorum*, *Fusarium oxysporum*, and *Fusarium verticillioides*) and biocontrol bacteria *B. velezensis* 20507 was cultured and activated at 28°C for 5 days in PDA medium. Five-millimeter plugs of plant pathogens were taken from actively growing cultures and inoculated into the center of the PDA plate, followed by *B. velezensis* 20507 (1 × 10^9^ CFU/mL, 20 μL) inoculation at four corners of the pathogen at a distance of 3 cm. Plant pathogen cultures without *B. velezensis* 20507 were used as the blank controls. Three biological replicates were used for each control and antagonistic plant-pathogen culture. After 5 days culture at 28°C, the growth of bacterial colonies was photographed and the inhibition rate was calculated using the following formula: Inhibition rate (%) = (radius of control colony × radius of treatment colony) × 100%/control colony radius.

### 4.7 Purification and activity detection of antifungal components

*Bacillus velezensis* 20507 was cultured in LB medium at 37°C at 200 r/min for 48 h. After centrifugation at 5000 × *g* for 10 min at 4°C, the fermentation broth was used for further antifungal substance extraction. Antifungal substances in the fermentation broth were purified using two methods: ammonium sulfate precipitation (1) and acid precipitation followed by methanol leaching (2). (1) Approximately 500 mL of fermentation liquid was poured into a 1 L triangular flask, and solid ammonium sulfate was slowly added to the flask until the ammonium sulfate saturation reached 10%. The solution was then placed in a magnetic stirrer and stirred overnight. Centrifugation was performed the next day at 5200 × *g* for 20 min at 4°C, the precipitation and supernatant were collected, respectively. More solid ammonium sulfate was added to the supernatant until saturation reached 20%. Thereafter, stirring and centrifugation were repeated to obtain crude protein with ammonium sulfate saturations of 20, 30, 40, 50, 60, and 70% in the fermentation broth. The precipitate was dissolved in 25 mmol/L Tris–HCl solution at pH 8.0, and a crude protein solution with a saturation of 10–100% ammonium sulfate in the fermentation broth was obtained. The obtained crude protein solution was dialyzed for 2 days to remove ammonium sulfate using a dialysis bag with a dialysis solution of 25 mmol/L Tris–HCl. (2) Approximately 500 mL of fermentation broth was poured into a 1 L triangular flask, and the pH was adjusted to 1.9 with concentrated hydrochloric acid. Then, the fermentation broth was placed in a 4°C refrigerator overnight. Centrifugation was performed the next day at 5200 × *g* for 20 min at 4°C to collect the sediment (Zhang and Sun, 2018), which was dried in a 60°C oven. The obtained solid matter was ground into a powder. Then, 250 mg of the powder was dissolved in 50 mL ddH_2_O to obtain a crude extract solution. The C18 solid-phase extraction column was activated sequentially with chromatography-grade methanol and ddH_2_O, and 30 mL of the solution was passed through the C18 solid-phase extraction column. The C18 column was then rinsed with 50, 60, 70, 80, 90, and 100% methanol and each eluent was collected. A rotary evaporator was used to removed water and methanol in the eluent at 40°C. The solid matter was dissolved in 4 mL 25 mmol/L Tris–HCl. The agar well diffusion method was used to determine the antagonistic activity of *B. velezensis* 20507 against *S. sclerotiorum.*

### 4.8 The destructive effect of antifungal substances on cell membranes

Five-millimeter plugs of *S. sclerotiorum* were obtained from an actively growing culture. The plugs were then inoculated into 6 cm diameter Petri dishes containing PDA medium. After being cultured for 7 days, 20 five-millimeter plugs were taken from the PDA medium and inoculated into 500 mL of potato dextrose broth (PDB), and cultured on a shaker for 48 h. Culture temperature and shaking speed were set as 28°C and 180 rpm. During the oscillation cultivation process, the mycelia of *S. sclerotiorum* intertwined to form mycelium balls with diameters of approximately 0.6 cm. Based on gradient processing pre-experiments, a small amount of mycelium was taken from the mycelium ball using needle-nose pliers, placed in 100 μg/mL ME80 aqueous solution for 20 min, and rinsed twice with PBS buffer. Mycelium soaked in distilled water for 20 min was used as the control. The fluorescent dye propidium iodide (PI, 2.5 μg/mL) dissolved in PBS was used to stain the mycelia, followed by rinsing twice with PBS to remove PI (Liu W. et al., 2022). The mycelia were observed under a Nikon Ti-S inverted fluorescence microscope at an excitation wavelength of 535 nm.

### 4.9 Purification and identification of antifungal substances

Full-wavelength scanning of antifungal substances in ME80 was performed using high-performance liquid chromatography (HPLC) to obtain the UV absorption peaks of each antifungal substance. The mobile phase was pure water, with a flow rate of 1 mL/min, a wavelength of 203 nm, and an injection volume of 50 μL. According to the peak-time graph, after the substance started to peak, the substance collection solution from 12 to 15 min was mixed to obtain component 1, and the substance collection solution from 21 to 23 min was mixed to obtain component 2. After freeze-drying, components 1 and 2 were dissolved in ddH_2_O for antagonistic performance testing. Substance 1 was subjected to Mass Spectrometry, and was commissioned to the Scientific Compass Analysis and Testing Center (Hangzhou, China).

### 4.10 Thermo stability evaluation

After sterilization at 121°C for 20 min, Potato Dextrose Agar (PDA) medium was prepared for evaluating the thermal stability of components 1 and 2 isolated from *B. velezensis*. Five millet plugs of *S. sclerotiorum*, taken from an actively growing culture, was inoculated in the center of the PDA medium and cultured for 2 days. Components 1 and 2 were dissolved in ddH_2_O to achieve a final concentration of 100 mg/L. Each component included two treatments: heat treatment (HT) and ordinary temperature treatment (CK). For HT, the substance to be tested is treated at 121°C for 20 min, which is also a condition for sterilization of PDA culture medium. Then, a punch was used to drill four holes with a diameter of 6 mm around the inoculation site in the culture medium, and 1CK, 1HT, 2CK, and 2HT was added to each of the four holes, respectively, with a volume of 50 μL for each hole. The cultures were maintained in a 25°C incubator and observed whether the heating treatment had an impact on components 1 and 2.

## Data availability statement

The datasets presented in this study can be found in online repositories. The names of the repository/repositories and accession number(s) can be found in the article/Supplementary material.

## Author contributions

YC: Writing – original draft. HL: Writing – original draft. HH: Writing – original draft. XH: Writing – original draft. ZW: Writing – original draft. XG: Writing – original draft. JL: Writing – original draft, Writing – review and editing.

## Footnotes

1
https://canu.readthedocs.io/en/latest/

2
https://github.com/lbcb-sci/racon

3
https://github.com/broadinstitute/pilon

4
http://quast.sourceforge.net/quast

5
http://circos.ca/

6
https://github.com/tseemann/barrnap

7
http://lowelab.ucsc.edu/tRNAscan-SE/

8
http://www.repeatmasker.org/

9
https://github.com/hyattpd/Prodigal

10
http://www.ebi.ac.uk/Tools/msa/muscle/

11
https://antismash.secondarymetabolites.org/#/start

## Supplementary material

Supplementary Table 1 Gene annotation.
